# Development of selective culture media for efficient isolation of *Avibacterium paragallinarum* from chickens

**DOI:** 10.1128/jcm.00311-25

**Published:** 2025-07-18

**Authors:** Mariela E. Srednik, Mostafa M. S. Shelkamy, Amro Hashish, Nubia R. De Macedo, Yuko Sato, Mohamed M. El-Gazzar, Orhan Sahin, Qijing Zhang

**Affiliations:** 1 Department of Veterinary Microbiology & Preventive Medicine, College of Veterinary Medicine, Iowa State University 70724 , Ames, Iowa, USA; 2 Department of Veterinary Diagnostic & Production Animal Medicine, College of Veterinary Medicine, Iowa State University 70724 , Ames, Iowa, USA; 3 Department of Avian and Rabbit Medicine, Faculty of Veterinary Medicine, Suez Canal University 110179, Ismailia, Ismailia Governorate, Egypt; University of California, Davis, Davis, California, USA

**Keywords:** *Avibacterium paragallinarum*, diagnosis, isolation, infectious coryza, chickens

## Abstract

Infectious coryza (IC) caused by *Avibacterium paragallinarum* (AvP) is an upper respiratory disease in chickens and incurs a significant economic impact on laying hens. Control of IC requires reliable bacterial isolation, but AvP is a fastidious bacterium, and the currently used methods yield poor isolation. To address this need, we developed selective media for the efficient growth and isolation of AvP. Several basal media supplemented with various growth factors were explored, and Mueller Hinton agar (MHA) supplemented with fetal bovine serum (FBS) plus NAD yielded optimal AvP growth, eliminating the need for nurse bacteria. This medium (named MSN) was further supplemented with vancomycin and crystal violet to produce two selective media, named MSNV and MSCV, respectively, to inhibit commensal Gram-positive bacteria that reside in the upper respiratory tract of chickens. MSNV and MSCV were compared with the standard isolation methods used in veterinary diagnostic laboratories for AvP isolation using samples from IC-positive and IC-negative flocks verified by clinical observation and AvP-specific RT-PCR. The two selective media significantly increased the isolation of AvP from clinical cases (59.4% for MSNV and 46.9% for MSCV) compared to the conventional method (37.7%). In addition, MSNV and MSCV successfully isolated the recently discovered non-pathogenic AvP variants, which were difficult to obtain using the standard methods. Notably, both media drastically reduced background bacteria and improved the purity of AvP isolates. These results demonstrate the efficacy and usefulness of the selective media for AvP isolation, which will enhance IC diagnosis, antimicrobial susceptibility testing, and vaccine development.

**IMPORTANCE:**

Infectious Coryza is an economically important disease in poultry, resulting in poor growth and a significant reduction in egg production. The causative agent (AvP) of the disease is difficult to isolate from clinical samples and often requires a nurse bacteria to provide essential nutrients for optimal growth. In addition, the currently used methods for isolation are inefficient and often result in contamination by residential bacterial species. The selective media (MSNV and MSCV) developed in this study solve these problems by eliminating the need for nurse bacteria and by inhibiting Gram-positive bacteria normally present in the respiratory tract of chickens. The media not only increase isolation efficiency but also improve the purity of the isolates. These advancements will facilitate IC diagnosis and the development of vaccines for effective control of this major poultry pathogen.

## INTRODUCTION

*Avibacterium paragallinarum* (AvP), previously known as *Haemophilus paragallinarum,* is a fastidious, slow-growing Gram-negative bacterial organism and is the etiological agent of infectious coryza (IC) (1). IC is an upper respiratory disease in chickens with clinical manifestations of nasal discharge, facial swelling, lacrimation, conjunctivitis, and anorexia (1, 2). Flocks infected by IC often experience a drop in feed and water consumption, followed by a drastic drop in egg production in layer chickens, resulting in significant economic losses to poultry producers (3). IC is historically reported worldwide, but is more prevalent in warm weather regions (4). However, in 2018, IC emerged in multiple Midwestern states, where the disease did not exist before. Since 2019, multiple IC outbreaks have been reported in broilers, layer pullets, and laying hens in Pennsylvania (5). Recently, in late May 2023, the regions densely populated with layers in eastern Ohio and western Indiana experienced an epidemic of IC for the first time (4). The outbreak spread through the layer population (approximately 25 million layers) in this area, causing respiratory disease with common signs of facial swelling and significant production losses. In addition to chicken, clinical IC was reported in other gallinaceous birds like pheasants, quails, and peacocks (6–9), suggesting a possible transmission of AvP between poultry flocks and wildlife. The recent rise of IC in the Midwest of the U.S. and other parts of the world highlights the importance of improving diagnosis and isolation of AvP for better control of this costly disease.

AvP is a fastidious bacterium and typically requires nicotinamide adenine dinucleotide (NAD) or its reduced form (NADH) for growth in culture media (3), although there are exceptions with some AvP isolates being NAD independent (10–13). AvP is commonly grown in an atmosphere with 5%–10% carbon dioxide and an optimal temperature range of 35°C–42°C (1, 3). Due to the need for NAD/NADH for growth, some bacteria excreting V-factor (NAD) are used as nurse bacteria to support the growth of AvP on culture media, where AvP demonstrates satellitic growth around the nurse bacteria (14). *Staphylococcus epidermidis* and *Staphylococcus hyicus* have been demonstrated to be suitable nurse bacteria for AvP growth (14, 15). Recently, *Staphylococcus chromogenes* was demonstrated to be an abundant residential bacterium facilitating the growth of AvP to high density and was also found to exacerbate the clinical disease caused by AvP in the respiratory tract of chickens (16).

Presumptive diagnosis of IC can be made based on history, clinical signs, and necropsy lesions; however, IC diagnosis confirmation requires bacterial isolation or direct detection using PCR. While PCR is commonly used in diagnostic labs, isolation and identification of AvP is required for further whole-genome characterization, antimicrobial susceptibility testing, inclusion in autogenous vaccines, and potential development of future AvP vaccines (2). In addition to the aforementioned specific requirements for isolation of AvP, the isolation from clinical cases is frequently encountered by the rapid overgrowth of commensals that are naturally present in the upper respiratory tract. This competitive growth often leads to contamination and significantly hampers the successful recovery of pure AvP isolates (17). Currently, several different media are used for the isolation of AvP from the chicken infraorbital sinus in research and diagnostic laboratories (2, 14, 18–21). There were reported methods that utilize brain heart infusion (BHI) agar supplemented with NAD for isolation of AvP (22), or tryptic soy agar (TSA) supplemented with NAD for isolation (2). Although these culture media supplemented with NAD may eliminate the need for nurse bacteria, they do not prevent the overgrowth of background commensals and are usually inefficient for AvP isolation from clinical tissues. In an early study, Terzolo et al. (23) described the use of a selective medium (Columbia blood agar supplemented with bacitracin, cloxacillin, and vancomycin) for AvP isolation, but the study did not report whether the selective medium improved the isolation. Currently, most veterinary diagnostic laboratories (VDLs) employ nurse bacteria for isolation of AvP from clincal samples (24, 25).

To improve the isolation efficiency of AvP from clinical cases, there is a need for a selective culture medium that does not require nurse bacteria and also inhibits the growth of background commensal bacteria. This study aims to develop selective media that are free of nurse bacteria and optimized for the efficient growth and isolation of AvP, in comparison with the standard AvP isolation methods currently utilized by VDLs.

## MATERIALS AND METHODS

### Culture media

Four basal media were evaluated in this study including Casman medium (Neogen, Fisher Scientific, East Lansing, MI), Mueller Hinton Agar (MHA, BD Difco, Franklin Lakes, NJ), Columbia agar base (Neogen, Fisher Scientific), and tryptic soy agar (TSA, BD Difco). Growth supplements added to the basal media included defibrated horse blood (HemoStat), laked horse blood (Thermo Fisher Scientific, Waltham, MA), fetal bovine serum (FBS, Cytiva, GE Healthcare Life Sciences, Marlborough, MA), and NAD^+^ (Sigma-Aldrich, St. Louis, MO). To prepare *S. chromogenes* supernatant, we incubated *S. chromogenes* (strain 2020070297) in Mueller Hinton broth (MHB, Becton Dickinson) for 24 h at 37°C and filtered the culture using a 0.20 µm filter (Corning, Corning, NY). The cell-free supernatant was collected for use. In total, nine culture media were initially evaluated in this study (Table 1). The Mueller Hinton agar +5% FBS +25 µg/mL (0.0025%) NAD^+^ medium was named MSN (MH + Serum + NAD^+^) in this study. The concentration selection of NAD^+^ was based on a previously published study (26). Inhibitors of Gram-positive bacteria added to the media included vancomycin at a concentration of 50 µg/mL and crystal violet at a concentration of 4 µg/mL. The MSN agar supplemented with vancomycin is named MSNV, while the MSN agar supplemented with crystal violet is named MSCV.

**TABLE 1:** Agar media evaluated for isolating AvP in this study^*a*^

Agar base	Supplements	References
Columbia agar base	7% equine hemolyzed blood	(18, 20)
Columbia agar base	5% FBS + NAD^+^(25 µg/mL)	This study
Casman media	7% equine hemolyzed blood	This study
Casman media	5% FBS + NAD^+^(25 µg/mL)	This study
Tryptic soy agar	7% equine hemolyzed blood	This study
Tryptic soy agar	5% FBS + NAD^+^(25 µg/mL)	(2, 16)
Tryptic soy agar	7% laked horse blood	This study
Mueller Hinton agar	5% FBS + NAD^+^(25 µg/mL)	This study (MSN agar)
Mueller Hinton agar	5% FBS +5% *S*. *chromogenes* supernatant	This study
Mueller Hinton agar	5% FBS + NAD^+^(25 µg/mL) + vancomycin (50 µg/mL)	This study (MSNV)
Mueller Hinton agar	5% FBS + NAD^+^(25 µg/mL) + crystal violet (4 µg/mL)	This study (MSCV)

^*a*^ FBS: fetal bovine serum; NAD^+^: nicotinamide adenine dinucleotide; equine hemolyzed blood: defibrated horse blood was added to melted agar base at a temperature above 56°C to allow lysis of blood in agar.

### Methods used for the isolation of AvP at Iowa State University (ISU) Veterinary Diagnostic Laboratory (VDL)

The ISU VDL used three different media for the isolation of AvP. These include blood agar containing 5% sheep blood (SBA, Remel, Thermo Fisher Scientific, Lenexa, KS), tryptic soy agar (BD Difco) containing 4% agar and 5% bovine blood (prepared at ISU VDL), and chocolate agar (Remel, Thermo Fisher Scientific). A nurse bacterium (*S. hyicus*) was added to all these media.

### Bacterial strains

*Av. paragallinarum* ATCC 29545 and an isolate derived from a clinical IC case submitted to ISU-VDL, *Av. paragallinarum* 2022076419, were used for initial testing of the different media. Other bacterial species used in this study included *Lactobacillus salivarius* LAB-7 isolated from broiler chicken cecal content, *Staphylococcus chromogenes* 2020070297 (used as a nurse bacterium and for supernatant preparation), *S. hyicus* (used as a nurse bacterium), and other genera members of the *Pasteurellaceae* family, including *Pasteurella multocida, Actinobacillus pleuropneumoniae, Haemophilus influenzae, Mannheimia haemolytica,* and *Glaesserella parasuis* derived from clinical cases by the ISU VDL.

### Clinical cases and samples

A total of 32 IC case submissions (400 samples) were processed using the MSNV and MSCV media in parallel with the VDL methods. Samples received were classified according to the presence or absence of clinical signs and real-time PCR (RT-PCR) test results (27). The cases were from Ohio, Arkansas, Indiana, and Pennsylvania. All cases were received and processed at the ISU VDL in 2023. The RT-PCR testing was performed to detect non-pathogenic and pathogenic AvP strains using the generic AvP-specific *rec*N assay (28) and the recently developed differential RT-PCR assays as previously described (27).

### Isolation of AvP from clinical samples

The infraorbital sinus of dead birds was swabbed with a sterile cotton swab after cutting the infra-orbital sinus with a scalpel and streaked on various culture media. The VDL culture plates were streaked using *S. hyicus* as a nurse bacterium, while MSNV and MSCV media were streaked without a nurse bacterium. All plates were incubated at 37°C with 5.2% CO_2_ for 48 h. Suspected AvP colonies were picked, purified as needed, and confirmed by matrix-assisted laser desorption/ionization time-of-flight mass spectrometry (MALDI-TOF) following the manufacturer’s instructions (Bruker, Billerica, MA).

### Growth curve analysis

We determined the growth curve of a clinical AvP isolate (strain 2022076419) using either BD Bacto tryptic soy broth or MSN broth. The inoculum was a fresh preparation of the AvP isolate (OD_600_ = 0.5), which was diluted 1:10 in each growth medium. The inoculated media were incubated at 37°C with 5.2% CO_2_. Samples were taken at 6, 12, 24, and 48 h to measure the colony-forming units (CFUs) using the drop method (29). The growth curve was performed in triplicate.

### Statistical analysis

McNemar’s test with Edwards corrections was used to analyze the isolation rates by the new selective media for isolation of AvP from clinical samples in comparison with the VDL methods. The calculations were performed manually in a 2 × 2 contingency table.

## RESULTS

### Comparison of various media for supporting AvP growth

Several basal agar media, including MHA, TSA, Casman Media, and Columbia agar, supplemented with different growth factors (Table 1), were tested in the presence or absence of nurse bacteria for the growth of pure cultures of AvP (ATCC 29545). In the presence of a nurse bacterium (*S. chromogenes*), most of the tested media lacking NAD^+^ showed satellitic growth surrounding the nurse bacterium (Fig. S1). In the absence of a nurse bacterium, MSN agar demonstrated the best growth of AvP as evidenced by the number and size of bacterial colonies (Fig. S1). Although Mueller Hinton agar with 5% FBS +5% *S*. *chromogenes* supernatant supported AvP growth, the colonies were much smaller compared with those on the MSN agar (data not shown). TSA and Columbia agar +FBS + NAD also showed growth of AvP, but the colonies were fewer and smaller (Fig. S1C and D). In Casman media + FBS + NAD, AvP did not show any growth (Fig. S1E). When a nurse bacterium was added to MSN, AvP did not show satellitic growth (Fig. S1B), in contrast to the medium with blood (7% lake horse blood or 7% equine hemolyzed blood) but without these two supplements (Fig. S1F through I).

The efficiencies of the media in supporting AvP growth were further analyzed using a quantitative method. A fresh AvP culture (strain 2022076419) was diluted to an OD_600_ of 0.01, and 10-fold serial dilutions were made. The dilution series was spotted onto various medium plates, which were incubated at 37°C with 5.2% CO_2_ for 48 h (Fig. S2). After 24 h of incubation, colonies were only visible on the MSN plates, but not on the other types of media tested in this study (results not shown). After 48 h of incubation, MSN agar showed the best growth (1 × 10^8^ CFU/mL) as judged by colony numbers and sizes (Fig. S2A), while no colony growth was observed on Casman medium (Fig. S2D). TSA +FBS + NAD had 8.25 × 10^7^ CFU/mL (Fig. S2B), and Columbia agar +FBS + NAD had 8 × 10^7^ CFU/mL (Fig. S2C). Although the colony counts were not significantly different from those on MSN agar, the colony sizes were visibly larger on MSN agar than on tryptic soy agar and Columbia agar (Fig. S2A through C). Regarding the different basal media supplemented with blood, TSA +7% lake horse blood was the only one that showed AvP colony growth, but the colony sizes were too tiny to count accurately (Fig. S2E). Other media supplemented with blood did not show any growth (Fig. S2F through H).

### Quantitative comparison in broth culture

**Fig 1:**
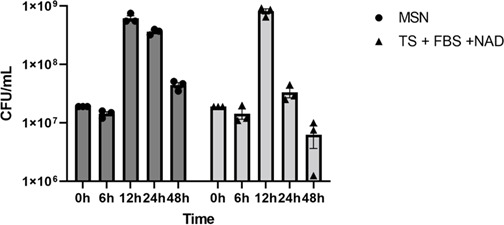
AvP growth dynamics in MSN broth (Mueller Hinton broth with 5% FBS and 25 µg/mL NAD) and tryptic soy broth with 5% FBS and 25 µg/mL NAD (TS + FBS + NAD). The same amount of AvP (strain 2022076419) was inoculated into each medium, and samples were collected at 6 h, 12 h, 24 h, and 48 h of incubation. CFU counts were determined using serial dilutions and plating on MSN (Mueller Hinton agar +5% FBS +25 µg/mL NAD) agar plates. The experiment was performed in triplicate.

We selected the two media that showed the best growth of AvP (MSN and TSA + 5% FBS +25 µg/mL NAD) to perform a growth curve analysis using their broth forms. The same number of AvP was inoculated into each broth medium, and CFUs/mL were measured at 6, 12, 24, and 48 h of incubation. With both media, significant growth was observed at 12 h. At 24 h, the AvP CFU remained high in MSN broth, but rapidly declined in the tryptic soy broth (Fig. 1), indicating that AvP died quickly in the latter medium once it entered the stationary phase.

### Development of selective media for AvP

The chicken upper respiratory tract is often colonized by Gram-positive commensal bacteria, which may overgrow AvP during isolation from clinical samples. To facilitate AvP isolation from clinical samples, we modified the MSN agar by the addition of vancomycin (MSNV) or crystal violet (MSCV). Both vancomycin and crystal violet are inhibitors of Gram-positive bacteria. We tested different concentrations of vancomycin (5, 20, 50, 100, and 200 µg/mL) and crystal violet (0.1, 0.5, 1, 2, and 4 µg/mL) in the MSN agar against a mixed culture of different bacteria (AvP, *S. chromogenes*, and *L. salivarius*) to determine the optimal concentrations that allowed AvP to grow but inhibited the background bacteria. With vancomycin, *S. chromogenes*, which are often present in the mucosa of the chicken respiratory tract, were inhibited at a concentration of 5 µg/mL. However, *L. salivarius,* another frequent contaminant in clinical samples, was not inhibited by vancomycin even at a concentration of 200 µg/mL, but was inhibited with 4 µg/mL of crystal violet (results not shown). Both selective media supported the growth of AvP without any noticeable inhibition on the size and number of AvP colonies. Consequently, we used 50 µg/mL vancomycin and 4 µg/mL crystal violet for supplementing the MSNV and MSCV media (Table 1), respectively.

When we cultured the other members of the *Pasteurellaceae* family (*Pasteurella, Actinobacillus, Haemophilus, Mannheimia, Glaesserella*), we observed that all the members grew well on the MSCV agar and the MSNV agar, except for *Glaesserella parasuis*, which did not grow on MSNV (50 µg/mL vancomycin), but grew on MSCV (4 µg/mL crystal violet) (Fig. S3). The result indicates that the selective media broadly support the growth of members of the *Pasteurellaceae* family.

### Isolation of AvP from clinical cases with MSNV and MSCV

**Fig 2:**
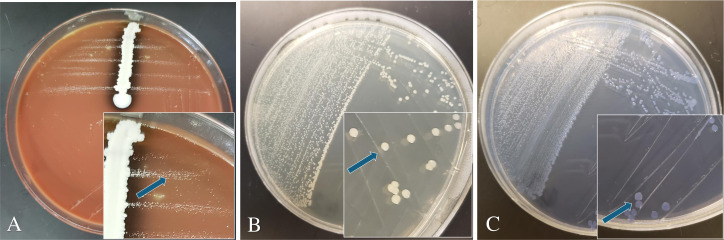
Isolation of AvP from a clinical sample with little background bacteria using three different culture media: chocolate agar with *Staphylococcus hyicus* as a nurse bacterium (**A**), MSNV agar (**B**), and MSCV agar (**C**). The three plates were inoculated with the same sample. MSNV: Mueller Hinton agar +5% FBS + 25 µg/mL NAD + 50 µg/mL vancomycin. MSCV: Mueller Hinton agar +5% FBS +25 µg/mL NAD + 4 µg/mL crystal violet. In each panel, the insert is an enlarged section of the same plate. Arrows indicate the growth of AvP colonies on each medium.

**Fig 3:**
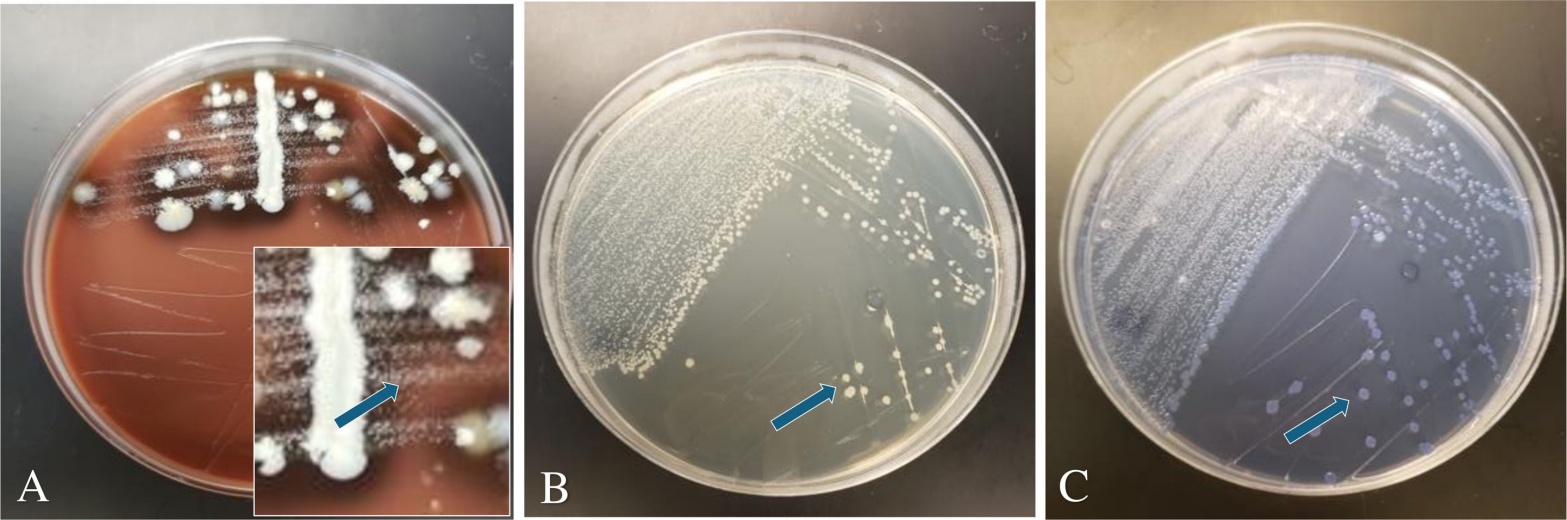
Isolation of AvP from a clinical sample moderately contaminated by background bacteria. The three plates are chocolate agar with *Staphylococcus hyicus* as a nurse bacterium (**A**), MSNV agar (**B**), and MSCV agar (**C**). The three plates were inoculated with the same clinical sample. Chocolate agar allowed significant background bacterial growth, while MSNV and MSCV yielded virtually pure AvP colonies. In panel A, the insert is an enlarged section of the same plate showing tiny AvP colonies close to the nurse bacterium. In all three panels, arrows indicate AvP colonies. MSNV: Mueller Hinton agar +5% FBS +25 µg/mL NAD + 50 µg/mL vancomycin. MSCV: Mueller Hinton agar +5% FBS +25 µg/mL NAD + 4 µg/mL crystal violet.

We then used the MSNV and MSCV agar plates for the isolation of AvP from clinical samples. This was done in parallel with the methods used by the ISU-VDL: chocolate agar, blood agar with 5% sheep blood, and Difco tryptic soy agar with 5% bovine blood, all of which were streaked with *Staphylococcus hyicus* as a nurse bacterium. Compared to chocolate agar plus a nurse bacterium, colony sizes on MSNV and MSCV were much larger (2–5 mm) than on chocolate agar, where AvP colonies appear as dewdrops (0.5–1 mm) (Fig. 2). Among the tested samples, 25 cases (207 samples) were positive with IC clinical signs and RT-PCR positive, 4 cases (153 samples) were negative with clinical signs but RT-PCR positive, 1 case (5 samples) was positive with clinical signs but negative on RT-PCR, and 2 cases (35 samples) were negative with clinical signs and RT-PCR detection. From the 207 chicken heads derived from birds with IC clinical signs and tested positive for AvP by RT-PCR, the combination of MSNV and MSCV media isolated AvP from 131 (63.3%) of the samples. By contrast, the VDL methods obtained 78 (37.7%) isolations from the same set of samples (Table 2). The isolation rates for MSNV and MSCV agar media differed slightly (Table S1), with MSNV yielding a higher isolation rate (*n* = 123, 59.4%) than MSCV (*n* = 97, 46.9%). Statistical analyses indicated that the isolation efficiency of either MSNV or MSCV was significantly higher than that of the current VDL methods (Tables S2 to S5; *P* < 0.001). In addition, the difference in isolation efficiencies between MSNV and MSCV was statistically significant (Table S5; *P* < 0.05). All of the isolates were confirmed to be AvP by MALDI-TOF. In addition to the increased isolation rate, the MSNV and MSCV greatly reduced the background bacterial contamination, making the task of obtaining a pure culture of AvP easier (Fig. 3).

**TABLE 2:** Overall performance of MSNV and MSCV for AvP isolation in comparison with the current ISU VDL methods^*e*^

No. samples^*a*^	IC signs^*d*^	RT-PCR^*b*^^,^^*d*^	Isolation by ISU VDL methods		Isolation by MSNV and MSCV	
			*Av. paragallinarum*	*Avibacterium* spp.	*Av. paragallinarum*	*Avibacterium* spp
207	+	+	78^*c*^	15	131^*c*^	18
153	−	+	0	1	4	6
5	+	−	0	3	0	2
35	−	−	0	0	0	11
**400**			**78**	**19**	**135**	**37**

^*a*^ Number of chicken head samples received at ISU VDL from chickens with or without clinical signs of Infectious Coryza.

^*b*^ Results of RT-PCR detection for AvP (27). ISU VDL = Iowa State University Veterinary Diagnostic Laboratory.

^*c*^ The isolation rates are significantly different (*P* < 0.001). MSNV = Mueller Hinton agar +5% FBS +25 µg/mL NAD + 50 µl/mL vancomycin. MSCV = Mueller Hinton agar +5% FBS + 25 µg/mL NAD + 4 µl/mL crystal violet. The total number for each column is shown at the bottom.

^*d*^ "+” indicates postive, while “−” depicts negative.

^*e*^ Bold formatting indicates the total number for each column.

**Fig 4:**
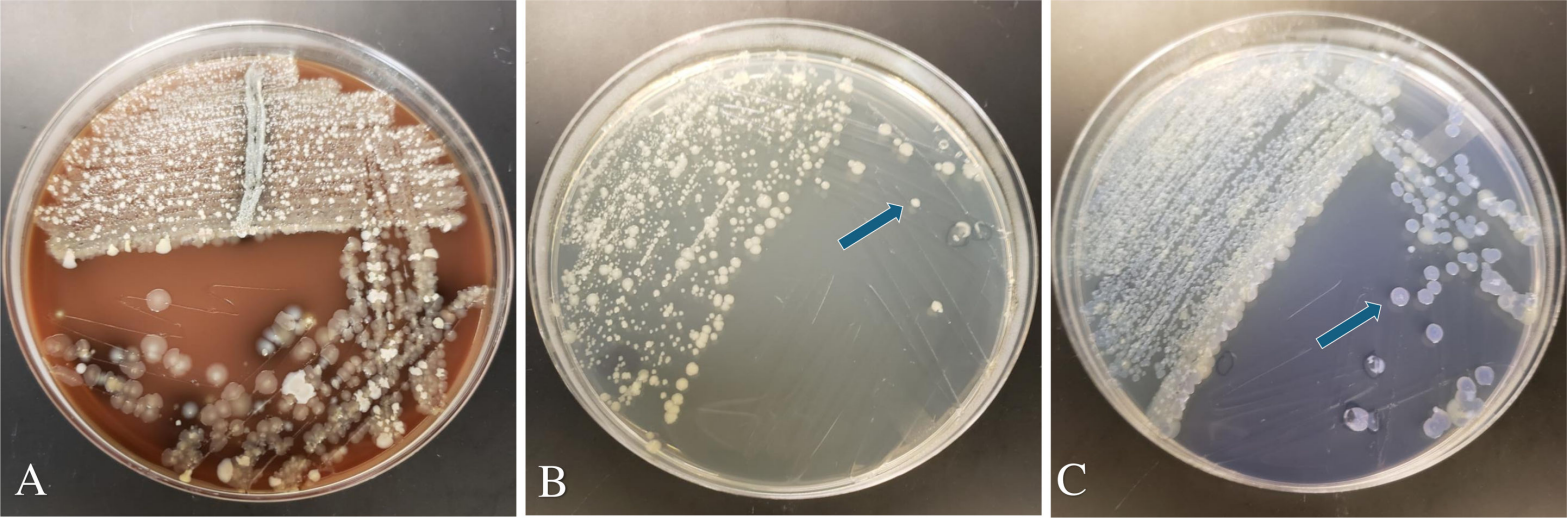
Comparison of different media for AvP isolation from a clinical sample heavily contaminated by background bacterial flora. The three used media are chocolate agar with *Staphylococcus hyicus* as a nurse bacterium (**A**), MSNV agar (**B**), and MSCV (**C**) agar. The three plates were streaked with the same clinical sample. Chocolate agar yielded heavy growth of background bacteria with no visible growth of AvP. MSNV and MSCV media showed mixed growth of AvP with some residential bacteria, but yielded much pure isolation of AvP. Arrows in panels B and C indicate AvP colonies, which appear in yellowish on MSNV agar and a light violet color on MSCV. MSNV: Mueller Hinton agar +5% FBS +25 µg/mL NAD + 50 µg/mL vancomycin. MSCV: Mueller Hinton agar +5% FBS +25 µg/mL NAD + 4 µg/mL crystal violet.

Although some clinical samples showed growth of Gram-negative background bacteria on MSNV and MSCV, AvP was distinguishable from other bacterial colonies based on size and morphology. With the MSCV media, AvP colonies showed a light violet color, while other background bacteria grew as yellowish colonies (Fig. 4).

### Isolation of non-pathogenic AvP using MSNV and MSCV

A non-pathogenic type of AvP (npAvP) was recently discovered in clinically normal flocks in the United States, which represents a unique variant of AvP (30). Although npAvP is detected by the RT-PCR, the organism was difficult to isolate from chicken samples. Using MSNV and MSCV, we isolated 4 npAvP from 153 chicken head samples that were derived from chickens with no IC signs but positive with the RT-PCR (Table 2). Notably, no npAvP from the chicken samples was obtained with the ISU VDL methods.

We also tested 5 samples from chickens with clinical signs of IC but AvP-negative RT-PCR and 35 samples from chickens without both IC signs and positive RT-PCR results. None of these samples yielded AvP isolation by the VDL methods or MSNV and MSCV (Table 2). Interestingly, from the 35 negative samples, the MSNV/MSCV agar plates isolated 11 *Avibacterium* spp. other than AvP, while the VDL method did not yield any *Avibacterium* spp. (Table 2).

By performing McNemar’s test in 2 × 2 contingency tables (Tables S2 to S5), we confirmed that the isolation results were significantly different between the methods. Specifically, we observed that the isolation of AvP using MSNV and MSCV in combination or either medium alone significantly increased the AvP isolation rate compared to the use of the conventional methods with nurse bacteria.

## DISCUSSION AND CONCLUSION

The fastidious nature of AvP and the high level of contamination of residential bacteria in the chicken upper respiratory tract require a selective method for cultivation and efficient isolation of AvP. In this study, we have developed two selective media, MSNV and MSCV, that do not require nurse bacteria and inhibit the growth of background commensal bacteria present in the samples. These two features led to significantly improved isolation and purification of AvP isolates from chicken clinical samples. From 207 clinical samples, the ISU VDL methods yielded a 37.7% isolation rate, while MSNV and MSCV obtained AvP from 63.3% of the samples. In addition, we also observed differences in the colony size, shape, and color that can help identify suspect AvP colonies. The use of MSNV and MSCV not only increased AvP isolation rates but also decreased the time for reporting from 4 to 2 days. This development represents a major improvement over the currently used methods for AvP isolation in diagnostic laboratories.

Among the basal media examined in this study, MSN showed the best support for AvP growth (Fig. 1; Fig. S1 and S2). Compared to the tryptic soy medium, which was commonly used in previously published studies (2, 16), AvP colonies on MSN agar were larger and visible after 24 h incubation. The use of MSN as broth also supported rapid growth of AvP, showing an exponential phase from 6 to 12 h, a stationary phase, and a smooth decline phase (Fig. 1). Although tryptic soy broth showed similar bacterial growth at 12 h, the viable bacterial counts decreased rapidly after that. At 24 h of incubation, the AvP CFU was more than 1-log higher in MSN broth than in tryptic soy broth (Fig. 1). This suggests that AvP died quickly in the tryptic soy broth once it entered the stationary phase. The result from broth cultures was also consistent with the growth observation of agar plates, which showed a higher number of colonies and bigger colony sizes on MSN agar than other agar bases (Fig. S2).

The MSN agar was further supplemented with vancomycin (MSNV) or crystal violet (MSCV) to inhibit Gram-positive resident bacteria, significantly enhancing the utility of the medium for isolating AvP from clinical samples. Compared to MSNV, MSCV yielded a lower AvP isolation rate from clinical samples (Table S1). However, MSCV helped to differentiate AvP colonies (light violet) from other bacterial contaminants (shown as yellow colonies) (Fig. 4). We also tested MSNV and MSCV for their suitability to support the growth of the other bacterial species in the *Pasteurellaceae* family. Most of the tested bacterial species, except for *Glaesserella,* demonstrated good growth on both media (Fig. S3). *Glaesserella parasuis* only grew on MSCV, but not on MSNV, suggesting it is susceptible to vancomycin. Since the *Pasteurellaceae* family encompasses genera and species that infect different animal species, the finding in this study suggests that MSNV and MSCV may be potentially used to isolate these bacterial pathogens from different animal species. Although selective media for the *Pasteurellaceae* family were previously reported (31–33), the development of MSNV and MSCV provides a valuable addition to the methods used for the isolation of these pathogenic species from clinical samples.

Recently, npAvP was detected by RT-PCR in clinically normal chicken flocks in the U.S. (27). The npAvP strains are difficult to isolate because they are overgrown by other bacteria, including pathogenic AvP. However, they are similar to AvP in terms of growth characteristics. Notably, the use of the new media developed in this study allowed us to obtain several npAvP strains, for which the standard ISU-VDL protocol failed to isolate (Table 1; Table S1). These npAvP isolates were all from commercial layer operations without any previous history of IC. The success in isolating npAvP will significantly improve the characterization of this unique AvP variant. The complete genome sequences of two previously obtained npAvP strains revealed some unique features, such as a lack of capsule production and an extra-long *HMTp210* hemagglutinin gene that is three times larger than that of the classical pathogenic AvP isolates (30). However, the genomic comparison was based on a small number of npAvP isolates, and the success in isolating npAvP with MSNV and MSCV should further facilitate phenotypic and genotypic characterization of npAvP.

Interestingly, five samples from chickens positive with IC clinical signs but negative with the RT-PCR were also negative for AvP isolation by all methods used in this study, indicating the clinical disease was unlikely to be related to AvP. The exact cause of the clinical signs was unknown, but other pathogens might be involved in IC-like disease. For example, swelling of the face and wattles could also be chronic fowl cholera, Newcastle disease, infectious bronchitis, avian influenza, avian metapneumovirus (swollen head syndrome), mycoplasmosis, and infectious laryngotracheitis. Thus, differentiation by specific PCR or isolation of AvP is necessary for definitive diagnosis of IC.

In conclusion, we developed two selective agar media (MSNV and MSCV) that considerably improved the isolation efficiency and purification of AvP from clinical samples in this study. Using these media, we also obtained npAvP isolates from chickens without clinical signs of IC, whereas the conventional culture media for AvP were not successful in the isolation. The selective media can be easily prepared in-house or commercialized for large-scale production, facilitating their use in clinical diagnosis of IC at various settings. Since these media also support the growth of AvP in high quantities in pure cultures, they may also be used for vaccine preparation, including autogenous vaccine production necessary for the control of AvP outbreaks. Successful isolation will also enable antimicrobial susceptibility testing, detailed genomic characterization, and pathogenic studies. Moreover, these media could also be adapted to isolate other bacterial pathogens (e.g., members of the *Pasteurellaceae* family) in the same family that infect various animal species. This possibility remains to be examined in future studies.

## SUPPLEMENTAL MATERIAL

Supplemental figures and tables jcm.00311-25-s0001.pdf Fig. S1 to S3, and Tables S1 and S2.

ASM does not own the copyrights to Supplemental Material that may be linked to, or accessed through, an article. The authors have granted ASM a non-exclusive, world-wide license to publish the Supplemental Material files. Please contact the corresponding author directly for reuse.
