# Supplementary material for: Development of selective culture media for efficient isolation of Avibacterium paragallinarum from chickens
Source: J Clin Microbiol. 2025 Jul 18;63(8):e00311-25. doi: 10.1128/jcm.00311-25 (PMC12345139; doi:10.1128/jcm.00311-25)
Supplement: Supplemental figures and tables — Fig. S1 to S3, and Tables S1 and S2. [file jcm.00311-25-s0001.pdf]

SUPPLEMENTAL MATERIALS

Development of selective culture media for efficient isolation of *Avibacterium paragallinarum* from chickens

Mariela E Srednik[1#], Mostafa M.S. Shelkamy[2,3], Amro Hashish[2], Nubia R. De Macedo[2], Yuko Sato[2], Mohamed M. El-Gazzar[2], Orhan Sahin[2], Qijing Zhang[1*]

[1]Department of Veterinary Microbiology & Preventive Medicine, College of Veterinary Medicine, Iowa State University, 1800 Christensen Drive, Ames, Iowa 50011, USA

[2]Department of Veterinary Diagnostic & Production Animal Medicine, College of Veterinary Medicine, Iowa State University, Ames, IA 50011, USA

[3]Department of Avian and Rabbit Medicine, Faculty of Veterinary Medicine, Suez Canal University, Ismailia 41522, Egypt

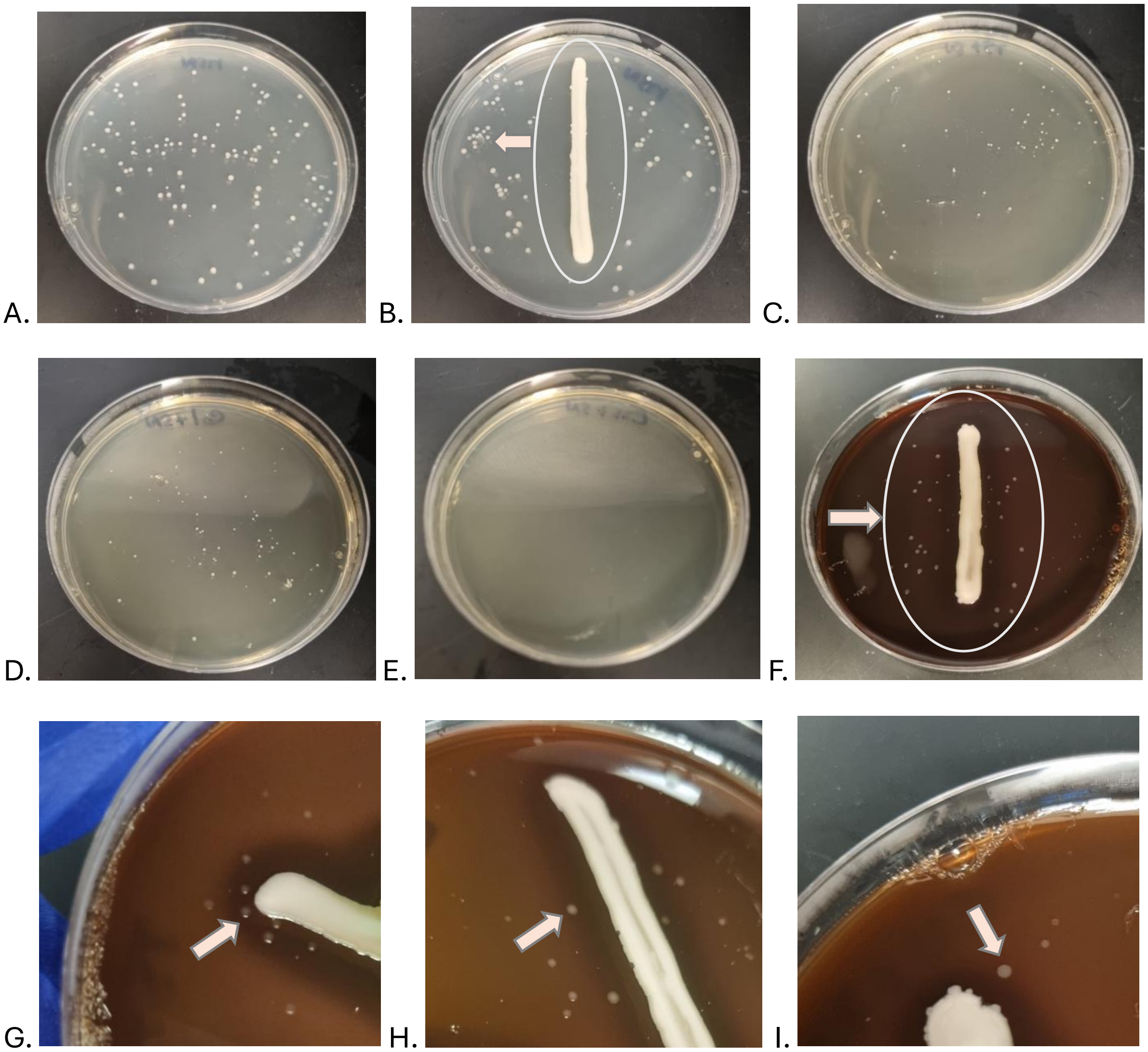

Suppl. Fig. 1. Growth of AvP on different culture media. Each plate was spread with the same dilution of a bacterial culture. On the blood agar media, *Staphylococcus chromogenes* was used as a nurse bacterium**. A.** MSN (Mueller Hinton Agar + Fetal Bovine Serum + NAD). **B.** MSN + nurse bacteria. **C.** Tryptic soy agar (TSA) + FBS + NAD. **D.** Columbia agar + FBS + NAD. **E.** Casman medium + FBS + NAD. **F.** TSA + 7% Lake Horse Blood + *S. chromogenes*. **G.** TSA + 7% Equine Hemolyzed Blood (EHB) + *S. chromogenes*. **H.** Columbia agar + 7% EHB + *S. chromogenes*. **I.** Casman medium + 7% EHB + *S. chromogenes*. For the media that were supplemented by FBS and NAD, the same concentrations of the supplements were used for all the media.

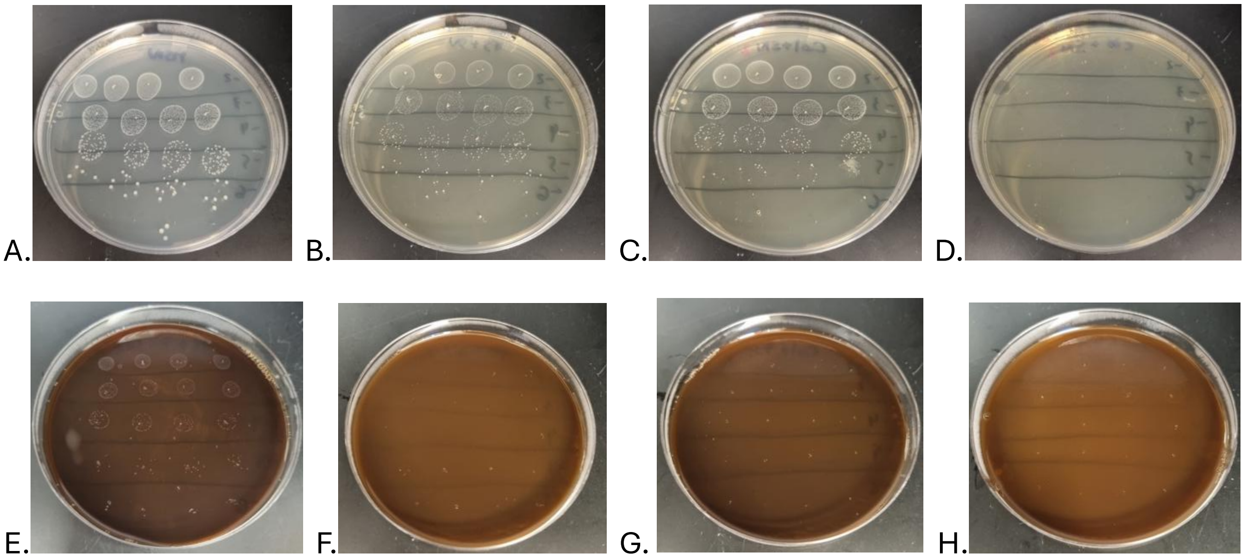

Suppl. Fig 2. Growth measurement of AvP growth in different culture media. A 10-fold dilution series of *Av. paragallinarum* ATCC 29545 was made in MSN broth and the -3, -4 -5 -6 dilutions were plated onto each medium using the drop method (4 drops/dilution). The plates were incubated at 37°C with 5.2% CO_2 for 48 h. **A.** MSN (Mueller Hinton Agar + Fetal Bovine Serum + NAD). **B.** Tryptic soy agar (TSA) + FBS + NAD. **C.** Columbia agar + FBS + NAD. **D.** Casman medium + FBS + NAD. **E.** TSA + 7% Lake Horse Blood. **F.** TSA + 7% Equine Hemolyzed Blood (EHB). **G.** Columbia agar + 7% EHB. **H.** Casman medium + 7% EHB. For the media that were supplemented by FBS and NAD, the same concentrations of the supplements were used for all the media.

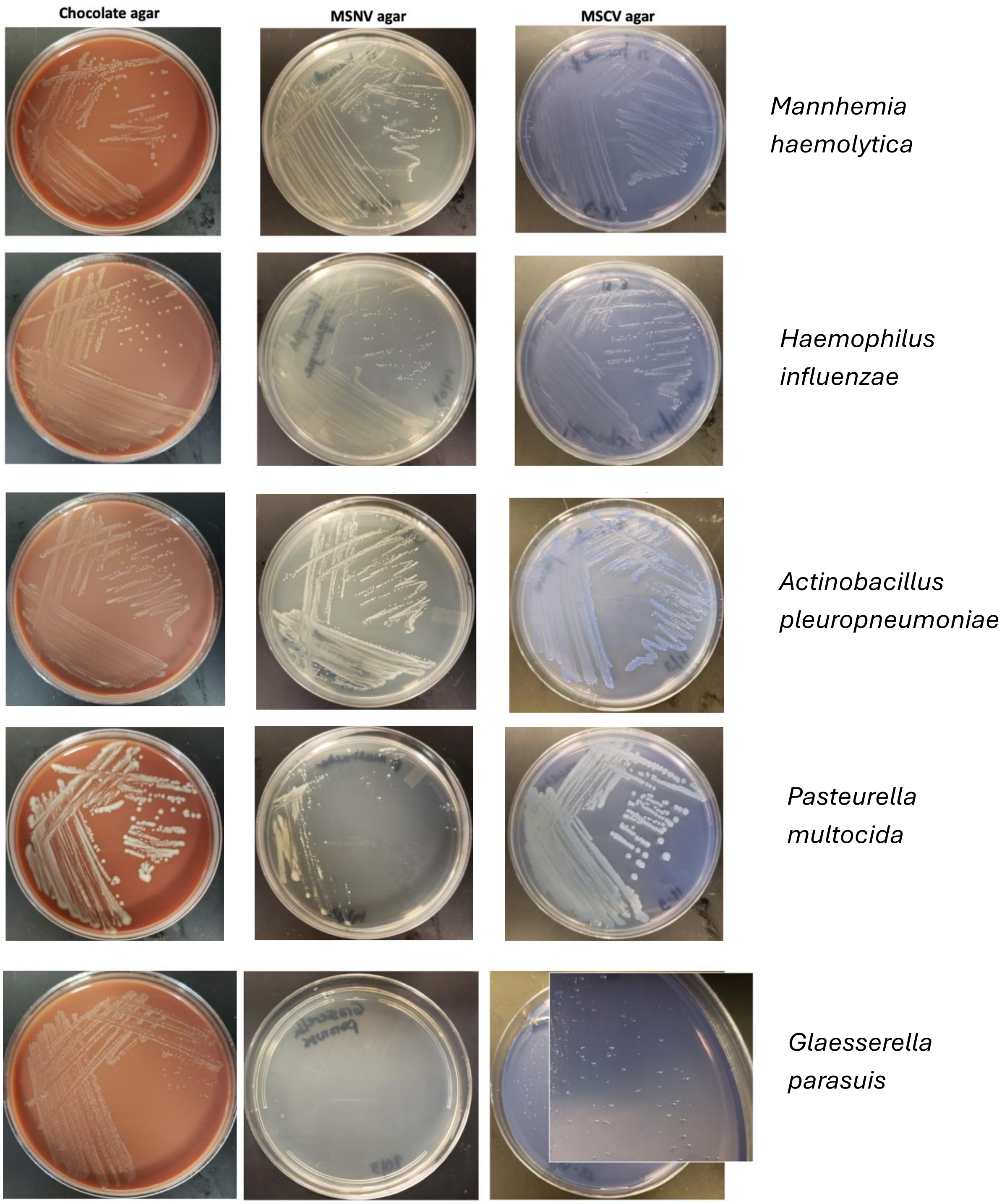

Suppl. Fig. 3. Growth comparison of different species of the *Pasteurellaceae* family on chocolate agar, MSNV agar, and MSCV agar. The names of the media are labeled on top of the panel, while the bacterial species are indicated on the right of the panel. *Glaesserella parasuis* is the species that does not grow on MSNV agar plate.

Suppl. Table 1. Performance of the selective media for isolation of AvP from clinical samples in comparison with the conventional VDL methods

**Case***	**IC signs**	**qPCR****	**VDL Methods**	**MSNV**	**MSCV**	**Both MSNV and MSCV**
10 chicken samples (2022119828)	+	+	**N=5 AvP**<br>N=1 *Av.* spp	**N=9 AvP**	**N=9 AvP**	**N=9 AvP**
10 chicken samples (2023013269)	+	+	N=0 (Negative)	N=0 (Negative)	N=0 (Negative)	N=0 (Negative)
15 chicken samples (2023028421)	+	+	N=0 (Negative)	N=3 *Av. endocarditis*	N=0 (Negative)	N=3 *Av. endocarditis*
5 chicken samples (2023029541)	+	+	N=0 (Negative)	N=0 (Negative)	N=0 (Negative)	N=0 (Negative)
3 chicken samples (2023044654)	+	+	**N=3 AvP**	**N=3 AvP**	**N=3 AvP**<br>N=1 *Av.* spp	**N=3 AvP**<br>N=1 *Av.* spp
6 chicken cases (2023044640)	+	+	**N=4 AvP**	**N=4 AvP**<br>N=1 *Av.* spp	**N=4 AvP**	**N=4 AvP**<br>N=1 *Av.* spp
5 chicken samples (2023048862)	+	+	**N=5 AvP**	**N=4 AvP**	**N=4 AvP**	**N=4 AvP**
7 chicken samples (2023050051)	+	+	**N=6 AvP**	**N=5 AvP**	**N=1 AvP**	**N=6 AvP**
6 chicken samples (2023050401)	+	+	N=3 *Av.* spp	N=3 *Av.* spp	N=2 *Av.* spp	N=3 *Av.* spp
6 chicken samples (2023050402)	+	+	**N=4 AvP**<br>N=1 *Av.* spp	**N=5 AvP**<br>N=2 *Av.* spp	**N=4 AvP**<br>N=3 *Av.* spp	**N=5 AvP**<br>N=3 *Av.* spp
6 chicken samples (2023051707)	+	+	**N=4 AP**	**N=5 AvP**<br>N=1 *A.* spp	**N=6 AvP**	**N=6 AvP**<br>N=1 *A.* spp
8 chicken samples (2023051732)	+	+	**N=6 AvP**	**N=7 AvP**	**N=7 AvP**	**N=7 AvP**
7 chicken samples (2023052510)	+	+	N=2 *Av.* spp	**N=3 AvP**<br>N=1 *Av.* spp	**N=4 AvP**	**N=4 AvP**<br>N=1 *Av.* spp
5 chicken samples (2023052689)	+	+	N=2 *Av.* spp	N=1 *Av. volantium*	N=1 *Av. volantium*	N=1 *Av. volantium*
12 chicken samples (2023052690)	+	+	**N=4 AvP**	**N=7 AvP**<br>N=3 *A.* spp	**N=7 AvP**<br>N=2 *A.* spp	**N=7 AvP**<br>N=4 *A.* spp
6 chicken samples (2023053534)	+	+	N=0 (Negative)	**N=6 AvP**	**N=6 AvP**	**N=6 AvP**
6 chicken samples (2023054185)	+	+	**N=3 AvP**	**N=5 AvP**<br>N=1 *Av.* spp	**N=4 AvP**	**N=5 AvP**<br>N=1 *Av.* spp
6 chicken samples (2023054115)	+	+	**N=3 AvP**	**N=5 AvP**	**N=6 AvP**	**N=6 AvP**
6 chicken samples (2023054116)	+	+	**N=4 AvP**	**N=5 AvP**<br>N=1 *Av.* spp	**N=5 AvP**<br>N=1 *Av.* spp	**N=5 AvP**<br>N=1 *Av.* spp
7 chicken samples (2023054117)	+	+	**N=4 AvP**	**N=7 AvP**	**N=7 AvP**	**N=7 AvP**
4 chicken samples (2023056904)	+	+	**N=2 AP**	**N=4 AvP**	**N=4 AvP**	**N=4 AvP**
6 chicken samples (2023058738)	+	+	N=0 (Negative)	N=0 (Negative)	N=0 (Negative)	N=0 (Negative)
7 chicken samples (2023058512)	+	+	**N=3 AvP**<br>N=1 *Av.* spp	**N=5 AvP**	**N=4 AvP**	**N=7 AvP**
10 chicken samples (2023058957)	+	+	**N=10 AvP**	**N=9 AvP**	**N=7 AvP**<br>N=1 *Av.* spp	**N=9 AvP**<br>N=1 *Av.* spp
38 chicken samples (2023064601)	+	+	**N=8 AvP**<br>N=5 *Av.* spp	**N=24 AvP**<br>N=2 *Av.* spp	**N=19 AvP**<br>N=2 *Av.* spp	**N=27 AvP**<br>N=2 *Av.* spp
**Total: 207 samples**			**N=78 AvP**<br>N=15 *Av.* spp	**N=123 AvP**<br>N=19 *Av.* spp	**N=97 AvP**<br>N=13 *Av.* spp	**N=131 AvP**<br>N=23 *Av.* spp

* Number of chicken head samples received at ISU VDL from chickens with clinical IC signs
**Results of qPCR detection specific for *Av. paragallinarum*

Suppl. Table 2. McNemar's test comparing the isolation rates of MSNV + MSCV and the VDL methods.

	**VDL* (P)**	**VDL* (N)**	**Total**
**MSNV+MSCV (P)**	70 (a)	61 (b)	131
**MSNV+MSCV (N)**	7 (c)	69 (d)	76
**Total**	77	130	207

P=positive, N= negative. McNemar's test with Edwards corrections: X^2= [(b-c)]^2 / b+c = 42.88, *p* value= 0.00000000058133

Suppl. Table 3. McNemar's test comparing the isolation rates of MSNV and the VDL methods.

	**VDL* (P)**	**VDL* (N)**	**Total**
**MSNV (P)**	70 (a)	54 (b)	124
**MSNV (N)**	7 (c)	76 (d)	83
**Total**	77	130	207

P=positive, N= negative. McNemar's test with Edwards corrections: X^2= [(b-c)]^2 / b+c = 36.21, *p* value= 0.000000001768762

Suppl. Table 4. McNemar's test comparing the isolation rates of MSCV and the VDL methods.

	**VDL* (P)**	**VDL* (N)**	**Total**
**MSCV (P)**	58 (a)	52 (b)	110
**MSCV (N)**	19 (c)	78 (d)	97
**Total**	77	130	207

P=positive, N= negative. McNemar's test with Edwards corrections: X^2= [(b-c)]^2 / b+c = 15.338, *p* value= 0.00008988872

Suppl. Table 5. McNemar's test comparing the isolation rates of MSNV and MSCV.

	**MSCV (P)**	**MSCV (N)**	**Total**
**MSNV (P)**	104 (a)	20 (b)	124
**MSNV (N)**	8 (c)	74 (d)	83
**Total**	113	94	207

P=positive, N= negative. McNemar's test with Edwards corrections: X^2= [(b-c)]^2 / b+c = 4.172, *p* value= 0.041087225
